# Magic-Factor 1, a Partial Agonist of Met, Induces Muscle Hypertrophy by Protecting Myogenic Progenitors from Apoptosis

**DOI:** 10.1371/journal.pone.0003223

**Published:** 2008-09-16

**Authors:** Marco Cassano, Stefano Biressi, Amanda Finan, Laura Benedetti, Claudia Omes, Renata Boratto, Frank Martin, Marcello Allegretti, Vania Broccoli, Gabriella Cusella De Angelis, Paolo M. Comoglio, Cristina Basilico, Yvan Torrente, Paolo Michieli, Giulio Cossu, Maurilio Sampaolesi

**Affiliations:** 1 Translational Cardiomyology, Stem Cell Institute Leuven (SCIL), KULeuven, Leuven, Belgium; 2 Department of Histology and Medical Embryology, University of Rome Sapienza, Rome, Italy; 3 Stem Cell Research Institute, H. S. Raffaele, Milan, Italy; 4 Human Anatomy, University of Pavia, Pavia, Italy; 5 Dompè Ph.r.ma. Research Center, L'Aquila, Italy; 6 Division of Molecular Oncology, Institute for Cancer Research and Treatment (IRCC), Candiolo (Torino), Italy; 7 Department of Neurological Science, Ospedale Maggiore Policlinico, University of Milan, Milan, Italy; Universität Heidelberg, Germany

## Abstract

**Background:**

Hepatocyte Growth Factor (HGF) is a pleiotropic cytokine of mesenchymal origin that mediates a characteristic array of biological activities including cell proliferation, survival, motility and morphogenesis. Its high affinity receptor, the tyrosine kinase Met, is expressed by a wide range of tissues and can be activated by either paracrine or autocrine stimulation. Adult myogenic precursor cells, the so called satellite cells, express both HGF and Met. Following muscle injury, autocrine HGF-Met stimulation plays a key role in promoting activation and early division of satellite cells, but is shut off in a second phase to allow myogenic differentiation. In culture, HGF stimulation promotes proliferation of muscle precursors thereby inhibiting their differentiation.

**Methodology/Principal Findings:**

Magic-Factor 1 (Met-Activating Genetically Improved Chimeric Factor-1 or Magic-F1) is an HGF-derived, engineered protein that contains two Met-binding domains repeated in tandem. It has a reduced affinity for Met and, in contrast to HGF it elicits activation of the AKT but not the ERK signaling pathway. As a result, Magic-F1 is not mitogenic but conserves the ability to promote cell survival. Here we show that Magic-F1 protects myogenic precursors against apoptosis, thus increasing their fusion ability and enhancing muscular differentiation. Electrotransfer of Magic-F1 gene into adult mice promoted muscular hypertrophy and decreased myocyte apoptosis. Magic-F1 transgenic mice displayed constitutive muscular hypertrophy, improved running performance and accelerated muscle regeneration following injury. Crossing of Magic-F1 transgenic mice with α-sarcoglycan knock-out mice –a mouse model of muscular dystrophy– or adenovirus-mediated Magic-F1 gene delivery resulted in amelioration of the dystrophic phenotype as measured by both anatomical/histological analysis and functional tests.

**Conclusions/Significance:**

Because of these features Magic-F1 represents a novel molecular tool to counteract muscle wasting in major muscular diseases such as cachexia or muscular dystrophy.

## Introduction

Hepatocyte Growth Factor (HGF), also known as Scatter Factor (SF), is a pleiotropic cytokine of mesenchymal origin that mediates a characteristic array of biological activities including cell proliferation, survival, motility and morphogenesis [1]–[3]. Its high affinity receptor, the tyrosine kinase Met, is expressed by a wide range of tissues including epithelial, endothelial, hematopoietic, neuronal and muscular cells [4], [5]. Embryonic muscle precursor cells express Met and migrate following HGF gradients during embryo development [6]–[10]. Genetic impairment of HGF-Met signaling in mice leads to abnormal muscle development in the limbs, thorax and tongue [11]–[13], and newborns -which are ataxic and have breathing problems- die a few hours later because they cannot suck mother's milk [14]. In the adult, the HGF-Met pathway is involved in muscle regeneration following injury. Muscle satellite cells, which reside in the stroma of muscular tissues and express both HGF and Met [15], represent a pool of muscle precursors that are activated and stimulated to divide when muscle regeneration or adaptive growth is needed [16], [17]. Autocrine HGF-Met stimulation plays a key role in mediating activation and early division of satellite cells, but is shut off in a second phase in order to allow the cells to exit the cell cycle and to enter the differentiation process [18], [19]. HGF stimulation of cultured satellite cells promotes cell proliferation and inhibits myogenic differentiation [20].

Magic Factor-1 (Met-Activating Genetically Improved Chimeric Factor-1 or Magic-F1) is an HGF-derived, engineered protein that contains two Met-binding domains repeated in tandem. It has a reduced affinity for Met and, in contrast to HGF, it elicits activation of the AKT but not the ERK signaling pathway. As a result of its partial ability to activate Met signaling, Magic-F1 is not mitogenic but conserves the ability to protect cells against apoptosis. We have analyzed the effects of Magic-F1 on muscular cells both *in vitro* and in mice. We show that Magic-F1 protects myogenic precursors against apoptosis and thus enhances the differentiation process, which is naturally accompanied by cell death. This pro-differentiative effect is observed both in cultured myogenic cell systems and in two different *in vivo* models. Remarkably, constitutive or transient expression of Magic-F1 in a mouse model of muscular dystrophy partially rescues the dystrophic phenotype and allows animals to perform better in a classic tread mill functional test. These features make Magic-F1 a novel, potential molecular tool to counteract muscle wasting in major muscular diseases including cachexia and muscular dystrophy.

## Results

### Engineering of Magic-F1, a bivalent Met ligand

**Figure 1:**
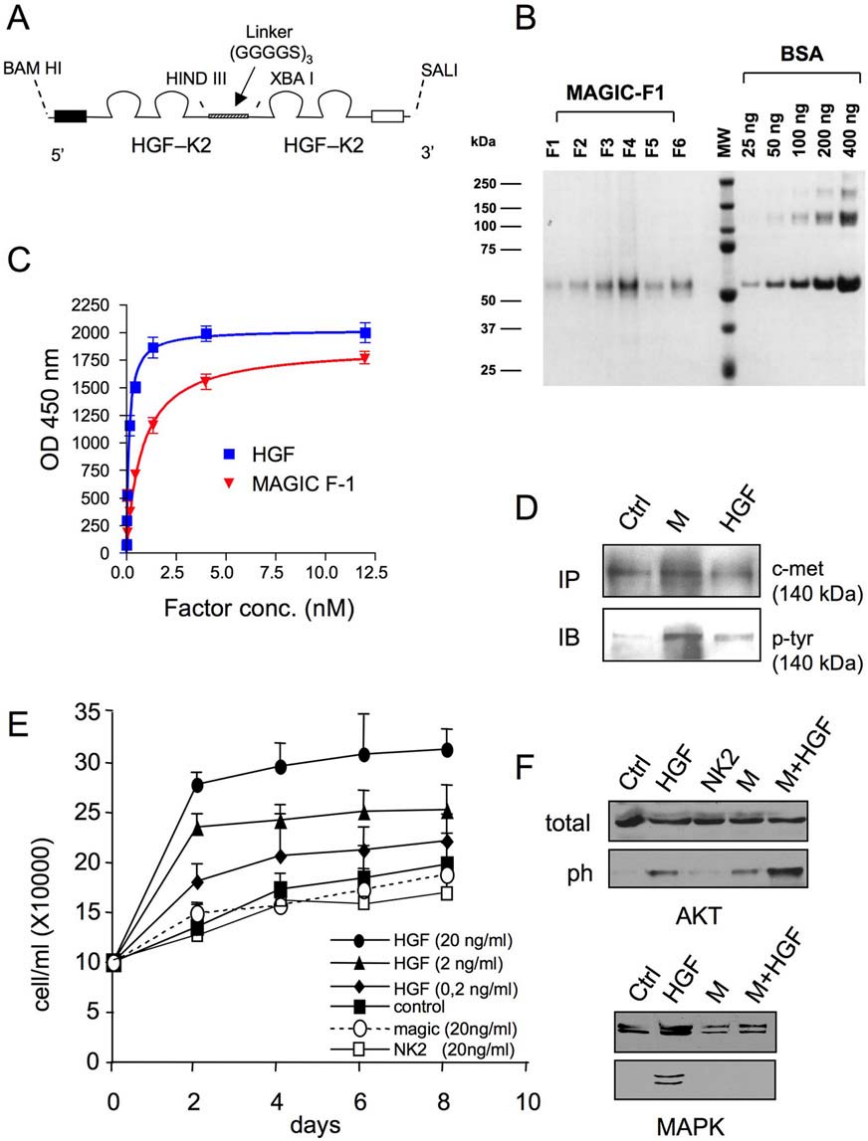
Magic-F1 elicits partial activation of the Met pathway in C2C12 myogenic cells. (A) Schematic representation of the Magic-F1 molecule. The indicated restriction sites refers to the corresponding cDNA map. *Bam*H1 and *Sal*1 were destroyed and the DNA fragment was cloned into the *Eco*RV restriction site of the pIRES-neo plasmid. (B) Purification of Magic-F1 by metal-chelate affinity chromatography. Following elution, fractions (F1-6) were resolved by SDS-PAGE in non-reducing conditions along with bovine serum albumine (BSA) standards. Proteins were revealed by Coomassie staining. MW, molecular weight; kDa, kilo Dalton units (C) ELISA binding assay. A fixed amount (100 ng/well) of Fc-Met chimera was absorbed in solid phase and exposed to increasing concentrations of HGF or Magic-F1 in liquid phase. Binding was revealed using biotinylated anti-HGF antibodies. (D) Met phosphorylation analysis in C2C12 cells. Cells were stimulated with no factor (Ctrl), 5 nM Magic-F1 (M) or 5 nM HGF (HGF), and Met phosphorylation was determined using anti-phosphotyrosine antibodies (IB). The same blots were reprobed with anti-Met antibodies to normalize the amount of receptor immunoprecipitated (IP). (E) Growth curves of C2C12 cells treated with the indicated concentrations of Magic-F1, HGF or no factor. (F) Signal transduction analysis. Cells were stimulated with no factor (Ctrl), 5 nM HGF, 5 nM NK2, 5 nM Magic-F1 (M), or 5 nM Magic-F1 and 5 nM HGF (M+HGF). Cell lysates were analyzed by Western blotting using antibodies against ERK or AKT (total) as well as antibodies against the phosphorylated forms of these signal transducers (ph).

Mature HGF is a dimeric molecule consisting of a α- and a β-chain joint by a disulphide bridge [21]. The α-chain contains a leader peptide for secretion, an N-domain similar to the activation domain of plasminogen, and four kringle domains (K 1–4) typical of the blood clotting cascade proteases [22]. In functional terms, HGF is a bivalent molecule containing two distinct Met binding sites, one in the α-chain high affinity; [23] and one at in the β-chain low affinity; [24]. Isolated HGF domains containing only one receptor binding site (HGF NK1, HGF NK2, HGF α-chain, HGF β-chain) can bind to the Met receptor but do not activate it [22]–[25], thus suggesting that a bivalent molecule is necessary to achieve receptor activation. Consistent with this idea, some monovalent scatter factor subdomains (HGF NK1, HGF NK2) display a partial agonistic activity when they are stabilized in a dimeric form by extracellular matrix proteoglycans [26]. To generate new recombinant proteins capable of inducing specific patterns of biological responses, we engineered several artificial molecules containing different HGF domain in various combination. Magic-F1, the prototype of this series, contains the signal peptide plus the N-domain and the first two kringles repeated in tandem and joint by a linker (Fig. 1A). A poly-histidine tag was engineered at the C-terminal end to facilitate protein purification. Since the high affinity Met binding site lies within the N and K1 domains [23], Magic-F1 is a bivalent ligand. Magic-F1 recombinant protein was produced using both transiently and stably transfected CHO cells, and was purified by affinity chromatography as described in the Experimental Protocol section (Fig. 1B). The affinity of Magic-F1 for Met was measured in a ELISA binding assay using a recombinant chimera between Met and the Fc portion of a human immunoglobulin Fc-Met; [27]. Fc-Met was absorbed in solid phase and exposed to increasing concentrations of Magic-F1 or HGF in liquid phase. Binding was revealed using biotinylated anti-HGF antibodies. This analysis revealed that Magic-F1 has an affinity for Met that is approximately 7–8 times lower than that of HGF (*i.e.* 0.8 nM; Fig. 1C). These data are consistent with previous measurements that determined the affinity of different subdomains of HGF for Met [23].

### Magic-F1 does not induce myoblast proliferation

Since HGF has been shown to affect satellite cell proliferation and differentiation, the action of the Magic-F1 on these biological processes was investigated by different approaches. We first subjected the myogenic cell line C2C12 [28] to different biological and biochemical assays in the presence of recombinant Magic-F1. Myoblast proliferation was evaluated by culturing C2C12 cells with Magic-F1, HGF or no factor as control. While HGF induced myoblast proliferation in a dose-dependent manner, Magic-F1 did not affect proliferation even at high concentrations as well as NK2 (Fig. 1E). As phosphorylation of Met is necessary for the activation of the HGF signaling cascade [1], we tested whether Magic-F1 could induce Met receptor phosphorylation. Immunoprecipitation analysis of Met followed by Western blot analysis using anti-phosphotyrosine antibodies revealed that both HGF and Magic-F1 induce phosphorylation of Met in C2C12 cells (Fig. 1D), indicating that the inability of Magic-F1 to affect myoblasts proliferation is not due to defective receptor activation. Since HGF is able to promote cell proliferation through the ERK pathway and to prevent apoptosis through AKT signaling [29], we next tested the ability of Magic-F1 to activate these two distinct pathways. While HGF induced phosphorylation of both MAPK and AKT. Magic-F1, differently form NK2, induced phosphorylation of AKT. Moreover, consistent with the idea that HGF and Magic-F1 compete for the same binding site on Met, Magic-F1 inhibited HGF-mediated MAPK phosphorylation (Fig. 1F).

### Magic-F1 promotes myoblast differentiation and survival

**Figure 2:**
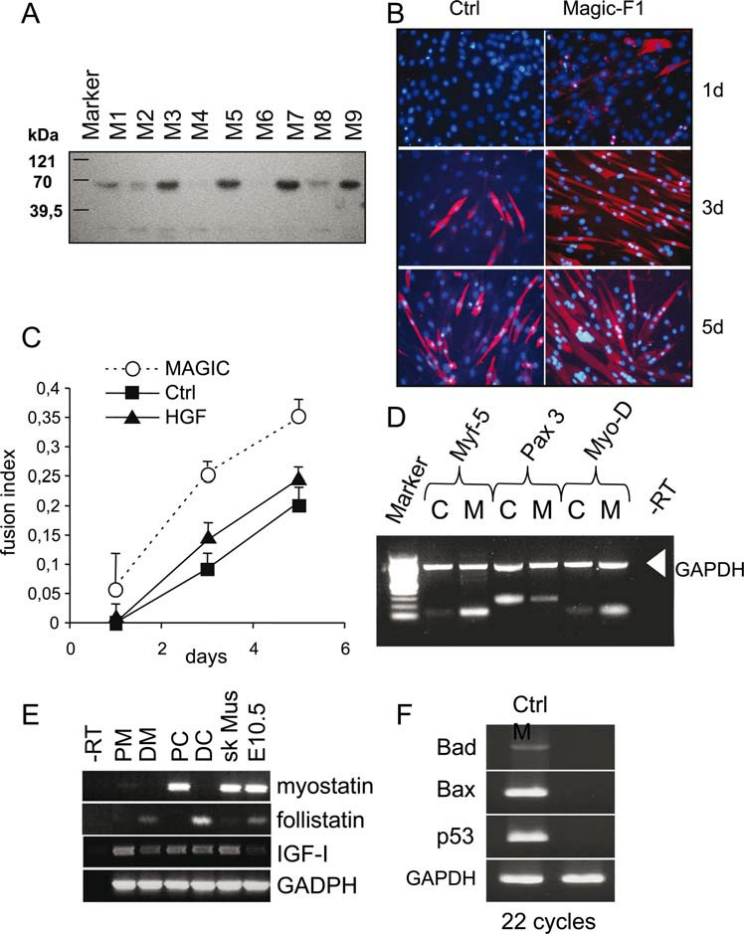
Morphological analysis of C2C12 cells expressing Magic-F1. (A) Magic-F1 detection in the culture media of stably transfected clones by Western blot analysis. (B) Immunofluorescence analysis for myosin heavy chain expression on Magic-F1 expressing clones (right panels) or control clones (left panels). Cells were analyzed after 1 day (1 d), 3 days (3 d) and 5 days (5 d). Nuclei were stained with DAPI. (C) Fusion index of C2C12 cells stably transfected with Magic-F1, HGF or mock-transfected (Ctrl). Fusion index is the ratio between the number of myocites with two or more nuclei versus the total number of myocites. (D) RT-PCR analysis of myogenic transcription factors (Myf-5, Pax3 and MyoD) on stably transfected clones (M) or control cells (C). GAPDH is used as internal control. (E) RT-PCR analysis of myostatin, follistatin and IGF1 expression in proliferating (P) versus differentiating (D) C2C12 cells. C, control cells; M, cells expressing Magic-F1. Skeletal muscle tissue (sk Mus) and a mouse embryo at 10.5 days (E10.5) were also used as controls. GAPDH was used as an internal control. (F) RT-PCR analysis of pro-apoptotic genes Bad, Bax and p53 in C2C12 clones stably transfected with Magic-F1 (M) or control cells (Ctrl). GAPDH was used as an internal control.

Next, we generated several stable clones of C2C12 myoblasts expressing Magic-F1 (Fig. 2A). Surprisingly, C2C12 cells expressing Magic-F1 differentiated at a faster rate compared to controls. In fact, they started to express myosin heavy chain, a marker of terminal differentiation, only one day following switch to differentiation medium (Fig. 2B). Consistent with accelerated differentiation, the myogenic markers MyoD and Myf5 were up-regulated while the Pax3 protein was down-regulated (Fig. 2D). Moreover, Magic-F1 increased the expression of 30 out of 36 genes known to be upregulated during C2C12 differentiation [30]; Figure S2. Magic-F1-expressing C2C12 cells fused into myotubes containing on average more nuclei than controls, while HGF did not affect myoblast fusion (Fig. 2C). Interestingly, in stable clones expressing Magic-F1, myostatin expression but not follistatin or IGF1 expression was down-regulated earlier compared to controls (Fig. 2E). This is in agreement with previous data showing promotion of myoblast differentiation and muscle hypertropy following myostatin ablation [31], [32]. Finally, cells expressing Magic-F1 displayed a marked reduction in the expression of several pro-apoptotic genes, including Bad, Bax and p53 (Fig. 2F) suggesting that the anti-apoptotic properties documented for HGF [29] are conserved in Magic-F1. Thus, Magic-F1 is an engineered, HGF-derived protein that elicits a selective pattern of biological responses on myoblasts. Firstly, it is a partial agonist of Met that activates the AKT pathway but not the ERK pathway. Secondly, it conserves the anti-apoptotic activity of HGF but not its mitogenic properties. Thirdly, it significantly enhances the differentiation potential of myoblasts without affecting their proliferation. The latter property is likely to be due to its inability to activate the ERK pathway.

### Electro-enhanced Magic-F1 DNA transfer in vivo promotes muscle hypertrophy and protects myocites against apoptosis

Efficient secretion of therapeutic proteins can be induced into skeletal muscle through electro-enhanced DNA transfer [33]. Using this technology, we tested the activity of Magic-F1 on mouse skeletal muscles *in vivo*. A plasmid encoding Magic-F1 was co-electroporated with a plasmid expressing β-galactosidase into the *tibialis anterior* and *quadriceps* muscles of juvenile mice (postnatal day 10) as described.

**Figure 3:**
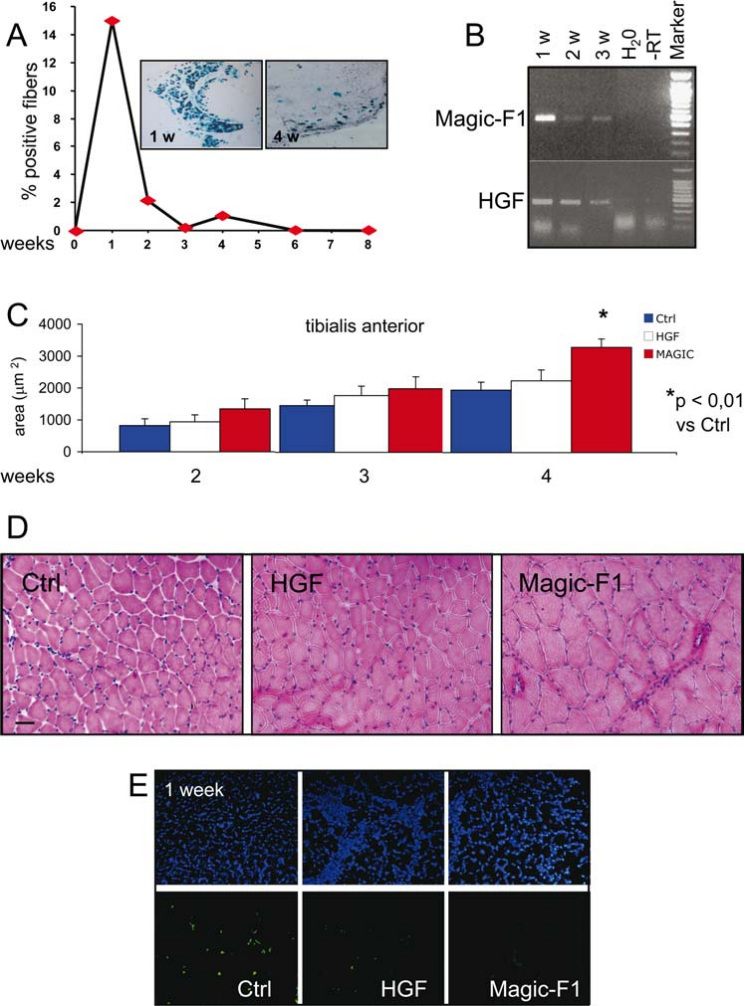
In vivo electrotransfer-mediated delivery of Magic-F1 to muscles. 10-day-old mice were subjected to *in vivo* electroporation of a combination of two plasmids expressing beta-galactosidase (β-gal) and HGF or Magic-F1, respectively. (A) Percent of β-gal-positive fibers following X-gal staining 4 weeks after electroporation. Representative images of electroporated muscles 1 week (1 w) and 4 weeks (4 w) after electrotransfer are shown in the upper panel. (B) RT-PCR analysis of electroporated muscles expressing HGF or Magic-F1 (data refers to samples obtained from *quadriceps*). (C) Histograms of morphometric analysis performed on *tibialis anterior*. Nine mice per group were analyzed. For each mouse, 300–460 fibers were examined (*p*<0.01). (D) H&E staining of *quadriceps* electroporated muscles. Note the larger fibers formed after 4 weeks in the Magic-F1 group relative to the control (Ctrl) or HGF group. (E) Tunel analysis of *quadriceps* 1 week after *in vivo* electrotransfer.

A vector encoding HGF and an empty vector without insert were used as controls. Histological analysis using X-gal staining showed that β-galactosidase was widely expressed one week after intra-muscular DNA electrotransfer but rapidly declined afterwards (Fig. 3A). Expression of the foreign genes also reached its maximum one week post-transfer and lasted for up to three weeks, as determined by RT-PCR analysis (Fig. 3B). Morphometric analysis performed on the *tibialis anterior* and *quadriceps* (9 mice for each group and 300–460 fibres for each sample were analyzed) revealed a significant increase of the cross-sectional area of Magic-F1-electrotransferred muscles compared to the control muscles starting two weeks after electrotransfer (Fig. 3C) as well as an increase in fiber perimeter (not shown). Representative images of electroporated *quadriceps* stained with hematoxylin and eosin are shown in Fig. 3D. Next, we evaluated whether Magic-F1 could protect muscle cells against apoptosis. To this end, we performed a TUNEL analysis of muscle sections one week after *in vivo* electrotransfer. This analysis indeed showed a decreased number of apoptotic nuclei (TUNEL positive) in muscles treated with either Magic-F1 or HGF (Fig. 3E). Taken together, the *in vitro* and *in vivo* data presented here suggest that Magic-F1 induces hypertrophy in the developing skeletal muscle by enhancing the differentiation and fusion ability of myogenic cells and by protecting them against apoptosis.

### Magic-F1 transgenic mice display hypertrophic fast-twitch fibers and improved running ability

**Figure 4:**
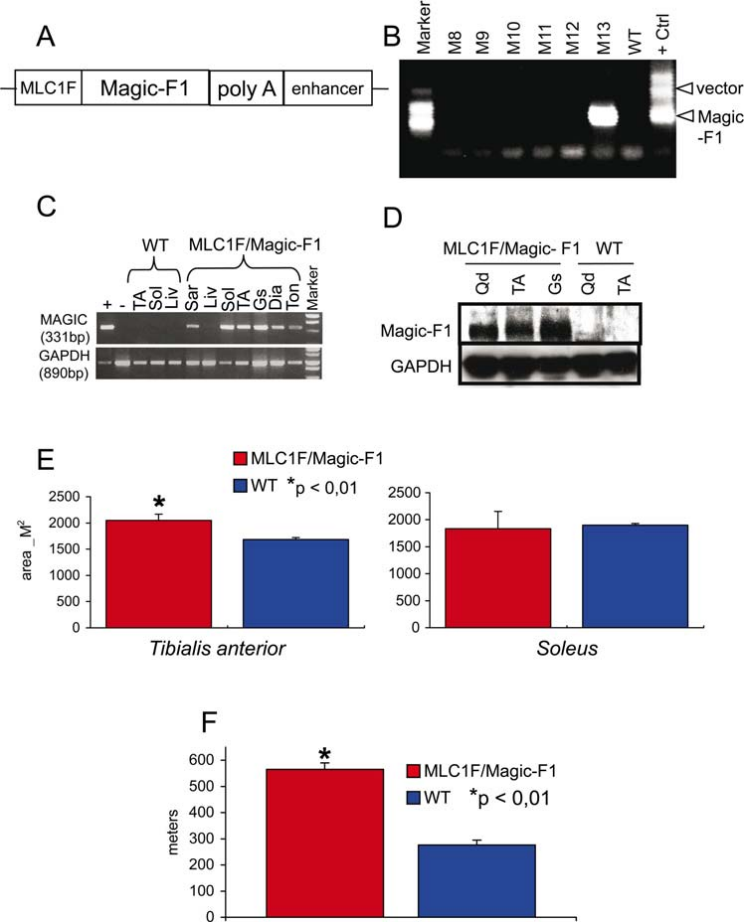
Morphological and functional analysis of Magic-F1 transgenic mice. (A) Transgenic construct used for two different microinjections into ES cells. (B) Representative example of tail genotyping by PCR. The M13newborn is positive for Magic-F1 integration. In two different microinjections we obtained 2 founders out of 14, that generated two different transgenic colonies. (C) RT-PCR analysis of Magic-F1 expression in transgenic (MLC1F/Magic-F1) and wild-type muscles (WT). No signal was detected in the liver of transgenic mice (Liv) or in any organ of wild-type animals. (D) Western blot analysis of Magic-F1 expression using anti-HGF antibodies or anti-GAPDH antibodies as control. A 60 kDa band appeared only in muscles from transgenic mice. (E) Morphometric analysis of *tibialis anterior* (left histogram) and *soleus* (right histogram) muscles. For each sample; 300–400 fibers were analyzed. (F) Distance performed by transgenic and control mice on a treadmill test. For more information, please refer to the Materials and Methods section.

To further investigate the ability of Magic-F1 to promote muscle hypertrophy, we generated transgenic mice expressing Magic-F1 under the control of the skeletal muscle-specific regulatory elements of the rat myosin light chain *MLC1F* gene locus [34]; Fig. 4A. MLC1F/Magic-F1 transgenic lines were identified by genotyping PCR with primers specific for the Magic-F1 coding sequence (Fig. 4B). Expression of the Magic-F1 transgene in adult mice was detected by RT-PCR in all muscles analyzed; on the contrary, no signal was detected in the liver of transgenic mice or in any organ of wild-type animals (Fig. 4C). Protein expression in fast transgenic muscles was also confirmed by Western blotting analysis (Fig. 4D). Embryonic and post-natal development of MLC1F/Magic-F1 transgenic animals occurred without overt differences compared to control mice. Skeletal muscle hypertrophy became apparent at around 5 weeks of age, consistent with the *in vivo* electrotransfer results. Morphometric analysis of the fast *tibialis anterior* muscles in transgenic mice showed a statistical significant increment of myofiber cross-sectional areas compared to age-matched wild-type controls (Fig. 4E, left histogram). Interestingly, morphometric analysis of slow-twitch *soleus* muscles unveiled no difference between transgenic and control animals (Fig. 4E, right histogram), even though transgene expression was detected in the *soleus* muscle (see Fig. 4C). A treadmill test was performed in order to evaluate the effect of Magic-F1 on muscular performance. This *in vivo* motility assay revealed that MLC1F/Magic-F1 transgenic mice cover on average a longer distance in comparison to their wild-type counterparts (Fig. 4F), thus demonstrating that Magic-F1-induced muscle hypertrophy results in increased muscular performance.

### Magic-F1 transgenic mice display enhanced muscle regenerative capacity

**Figure 5:**
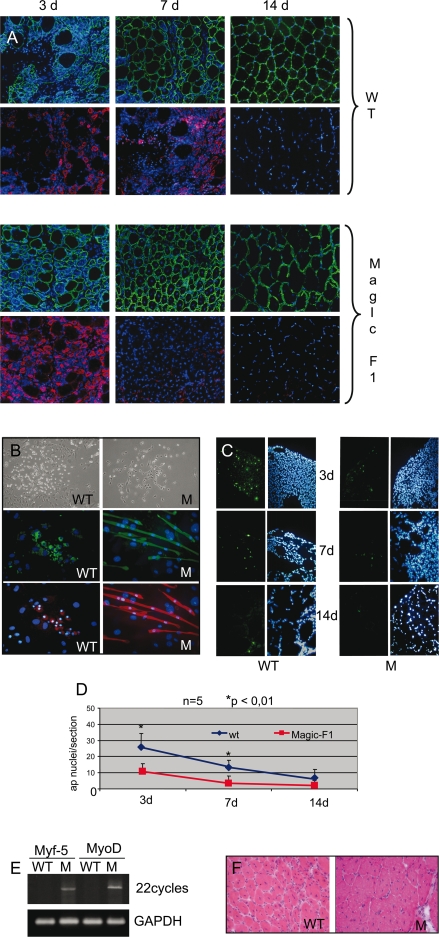
Magic-F1 promotes muscular regeneration. (A) Immunofluorescence analysis of muscle fibers using antibodies against embryonic myosin heavy chain (red) or laminin (green) in the *tibialis anterior* of transgenic and wild-type mice. Nuclei were stained with DAPI. (B) Immunofluorescence analysis for desmin (middle panels, in green) and myosin heavy chain (lower panels, in red) of satellite cells isolated from *tibialis anterior* of Magic-F1 transgenic mice (M) and wild-type (WT) mice subjected to cardiotoxin treatment. Nuclei are stained with DAPI (in blue). The upper panels show a phase contrast image of satellite cell clones, 3 days after low density seeding. (C) TUNEL analysis of *tibialis anterior* after 3, 7 and 14 days after cardiotoxin treatment. (D) Quantification of apoptotic nuclei (ap nuclei) relative to the experiment described in C. Red line, transgenic mice; blue line, wild-type mice. (E) RT-PCR analysis of myogenic transcription factor expression (MyoD and Myf5) conducted on *tibialis anterior* from transgenic (M) or wild-type (WT) mice. (F) Representative images of *tibialis anterior* muscles stained with H&E extracted from Magic-F1 transgenic mice and wild-type mice 10 days after cardiotoxin treatment. Note the larger size of fibers in the Magic-F1 group (M) compared to the control group (WT).

In muscular dystrophy disorders, fiber degeneration is only partially counterbalanced by regeneration of new fibers by satellite cells [35]. Hypertrophic factors represent a potential therapeutic approach against muscle wasting. We therefore analyzed the effect of Magic-F1 on muscle regeneration. Muscle damage was induced in the *tibialis anterior* muscles of adult MLC1F/Magic-F1 transgenic or wild-type mice by a single intramuscular injection of cardiotoxin. MLC1F/Magic-F1 transgenic animals responded to muscle crush by rapidly activating the regenerative program. Three days after cardiotoxin injection, an enhanced number of centrally-nucleated regenerating myofibers and an increased expression of the regeneration hallmark protein, embryonic myosin heavy chain (MyHC), was observed in damaged muscles of transgenic mice compared to those of age-matched wild-type animals (Fig. 5A). Furthermore, one week post-injury, muscle fibers of MLC1F/Magic-F1 transgenic mice were characterized by enhanced peripherycal localization of nuclei and by the down-regulation of embryonic MyHC, indicating successful completion of the regeneration program. In contrast, in wild-type animals, regeneration persisted for a few more days (Fig. 5A). Interestingly, also regenerating centrally-nucleated fibers in the MLC1F/Magic-F1 transgenic mice appeared to have a greater cross-sectional area in comparison to wild-type animals after 3 days of injury (Figure S3). Consistent with these observations, satellite cells collected from MLC1F/Magic-F1 transgenic showed enhanced differentiation potential *in vitro* compared to satellite cells from wild-type mice. Furthermore, satellite cells from MLC1F/Magic-F1 transgenic mice were more differentiation-prone as revealed by smaller clone size and accelerated appearance of differentiated myotubes (Fig. 5B). Moreover, cardiotoxin induced a rapid apoptotic response in injected areas, which appeared to be strongly reduced in MLC1F/Magic-F1 transgenic animals (Fig. 5C and D). Rapid and efficient muscle regeneration in transgenic muscles subjected to cardiotoxin treatment is also explained by earlier and increased expression of the muscle master genes *MyoD* and *Myf5* (Fig. 5E). This resulted in reduction of central nucleated fibers at 10 days following cardiotoxin treatment (Fig. 5F) and in greater cross-sectional area of regenerated transgenic fibers compared to wild-type animals (Figure S3B).

### Magic-F1 partially rescues the dystrophic phenotype of alpha-sarcoglycan knock-out mice

**Figure 6:**
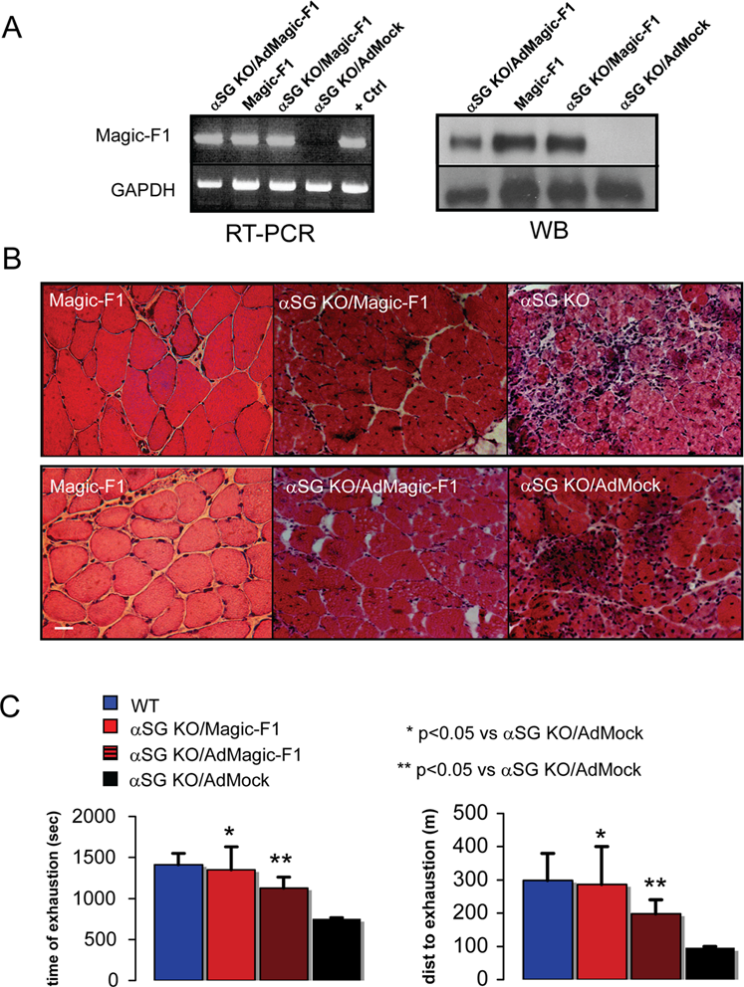
Magic-F1 increases muscle strength in α-SG knock-out mice. (A) After performance of an exhaustion treadmill tests, mice were sacrificed and Magic-F1 expression was evaluated by RT-PCR (left panel, upper lanes) and Western blot analysis (right panel, upper lanes) on *tibialis anterior* muscles of the indicated mice. Lane 1, α-SG knock-out dystrophic mice injected with an adenovirus expressing Magic-F1; lane 2, Magic-F1 transgenic mice; lane 3, α-SG knock-out/Magic-F1 transgenic mice; lane 4, α-SG knock-out dystrophic mice injected with an empty adenovirus. GAPDH was used as internal control (bottom panel). (B) H&E staining of *tibialis anterior* (left upper panel) and *quadriceps* (left lower panel) muscles from 3 month-old Magic-F1 mice, *tibialis anterior* of double transgenic α-SG knoc-out/Magic-F1 transgenic mice (upper middle panel), α-SG knock-out mice (right upper panel), α-SG knock-out mice injected with Ad-Magic-F1 (middle lower panel) or with Ad-Mock (right lower panel). Mice were sacrificed 14 days after the exhaustion treadmill tests. The bar is a 50 µm marker. (C) Exhaustion treadmill tests carried on wild-type mice (blue label), α-SG knock-out/Magic-F1 transgenic mice (red label), α-SG knock-out mice injected with Ad-Magic-F1 (red-black label) or Ad-Mock (black label). Note that mice expressing Magic-F1 showed increased running performance compared to Ad-Mock-injected mice. Muscle strength of treated and control animals was measured as time (left histogram) or distance to exhaustion (right histogram; n = 4, *p*<0.05).

The therapeutic potential of Magic-F1 was tested in alpha-sarcoglycan (α-SG) knock-out mice, which represent an established animal model of muscular dystrophy. Due to their genetic defect, these mice display persistent degeneration and regeneration areas in skeletal muscles [36]. To achieve Magic-F1 expression in these mice, we undertook two different approaches. Firstly, we crossed Magic-F1 transgenic mice with α-SG knock-out animals, thus generating α-SG knock-out mice expressing Magic-F1 in their muscles (Fig. 6A). Secondly, we engineered an adenoviral vector [37] expressing Magic-F1 and administered it by intramuscular injection to 45 day-old α-SG knock-out female mice under immunosuppressive conditions [38]. Morphological analysis of the *tibialis anterior* of α-SG knock-out/Magic-F1 transgenic mice revealed significant muscular hypertrophy compared to α-SG knock-out controls, which persisted until at least 6 months of age (Fig. 6B). Consisted with this, α-SG knock-out/Magic-F1 transgenic mice performed much better than control α-SG knock-out mice in a classic treadmill test (Fig. 6C). Adenovirus-mediated delivery of Magic-F1 also ameliorated the dystrophic phenotype of α-SG knock-out mice, although to a reduced extent compared to α-SG knock-out/Magic-F1 transgenic mice (Fig. 6C). This may be due to the lower expression levels of Magic-F1 achieved by adenoviral transduction (see Western blot analysis in Fig. 6A). In any case, the values obtained were statistically significant compared to dystrophic animals treated with a control adenovirus (Fig. 6C).

## Discussion

Protein engineering allows creating recombinant factors displaying selective biological functions. This is particularly useful for pleiotropic factors eliciting several different biological responses like HGF. Magic-F1, an engineered protein derived from HGF, maintains the ability to protect cells against apoptosis and to promote myoblast differentiation, but is devoid of any mitogenic activity typical of its parental factor. This results in remarkable enhancement of skeletal muscle regeneration without induction of cell proliferation, a crucial feature for its potential therapeutic application. Notably, muscle hypertrophy was induced in normal and regenerating muscle both when Magic-F1 was present as a transgene and when it was delivered to post-natal muscles, as it would occur in a cell or gene therapy context.

The potential relevance of inducing muscle hypertrophy to the treatment of muscle disorders in humans has been suggested by studies involving *mdx* mice, which carry a mutation in the dystrophin gene and therefore serve as a genetic model of Duchenne's muscular dystrophy [39]. For example, *mdx* mice lacking myostatin were found not only to be stronger and more muscular than their *mdx* counterparts with normal myostatin, but also to have reduced fibrosis and fat deposition, suggesting sustained muscle regeneration [40]. Furthermore, injection of neutralizing monoclonal antibodies directed against myostatin into either wild-type or *mdx* mice increases muscle mass and specific force, suggesting that myostatin plays an important role in regulating muscle growth in adult animals [41]. Magic-F1 is a molecule with a potential clinical application as it can induce muscle hypertrophy by both down-regulating myostatin and directly activating MyoD, Myf5 and several anti-apoptotic pathways. Interestingly, no side effects have been observed in skeletal muscles following electro-enhanced Magic-F1 DNA transfer or in transgenic mice expressing the Magic-F1 under the control of a muscle-specific promoter.

Our data showing the inability of Magic-F1 to induce the ERK pathway together with an inhibitory interference with HGF-induced ERK activation are particular relevant to a potential therapeutic use of this engineered factor. In fact, several tissues other than myocytes and satellite cells express the Met receptor, including epithelial cells of kidney, liver, lung, skin, breast and the whole gastrointestinal tract, as well as neurons, endothelial cells and hematopoietic precursors [1], [5]. Furthermore, Met overexpression is a very frequent event in human cancer [42]. This raises the concern that stimulating the proliferation of Met-expressing cells may lead to tumor formation or progression [43]. In this regard, the lack of any mitogenic activity makes Magic-F1 a potentially safe cytokine for cell therapy.

Because of its potent and selective effect on myoblast survival and differentiation, Magic-F1 promoted muscular hypertrophy in all mouse models analyzed. This biological activity, revealed by *in vitro* experiments, was extensively confirmed by the analysis of muscles treated by electro-enhanced DNA transfer or derived from transgenic mice expressing Magic-F1 under the control of a muscle-specific promoter. Interestingly, a statistically significant increase of myofiber cross-sectional areas was observed in the *tibialis anterior* muscles but not in slow-twitch *soleus* muscles. This can be attributed to the specificity of the promoter, active in fast twitch fibers, and to the fact that the amount of circulating Magic-F1 (escaping from fast-twitch muscles) is not enough to induce muscle hypertrophy. Moreover, following cardiotoxin treatment, regenerating centrally-nucleated fibers in the MLC1F/Magic-F1 transgenic mice appeared to have a greater cross-sectional area compared to wild-type animals. This can be explained by the enhanced differentiation potential of satellite cells, which indeed displayed an earlier differentiation program *in vitro* compared to cells isolated from wild-type mice. We previously reported the presence of myogenic precursors, named mesoangioblasts, in the skeletal muscles of mice [44], dogs [45] and humans [46]. These cells could also be positively affected by Magic-F1 and we cannot exclude their participation in the regeneration of skeletal muscle tissues. On the other hand, the rapid apoptotic response in cardiotoxin-treated muscles is strongly reduced in MLC1F/Magic-F1 transgenic mice. This results in a more evident muscular hypertrophy of transgenic muscles.

Several authors have reported that HGF inhibits muscle differentiation both *in vitro* and *in vivo* [19], [20]. Recently, it has been reported that HGF gene therapy improves LV remodeling and dysfunction post-infarction through promotion of cardiomyocyte hypertrophy, and that HGF plays a role in the induction of stem cell commitment to the cardiomyocyte lineage [47]–[49]. Magic-F1 exhibits biological effects in the renewal of skeletal muscles tissues similar though not identical to those observed for HGF in cardiac tissue regeneration. Further studies are necessary to elucidate the different potential effects of HGF in this context and –in this sense– supplementary studies on Magic-F1 signal transduction could provide useful information.

Successful adenovirus-mediated gene delivery under immunosuppressive conditions in adult muscles was previously demonstrated [50], [51]. In the present study, we transduced muscle fibers of juvenile α-SG knock-out mice with adenoviral vectors carrying Magic-F1 cDNA. All injected mice showed a physiological benefit and performed much better compared to mock-treated dystrophic animals in treadmill tests. As discussed, the less efficient rescue of the dystrophic phenotype by adenovirus-mediated Magic-F1 delivery compared to the crossing with Magic-F1 transgenic mice is conceivably due to incomplete muscle transduction. Importantly, in those mice in which all dystrophic fibers were transduced, the treadmill test performance was similar to that covered by control, non-dystrophic animals (not shown).

In conclusion, Magic-F1 is a soluble, engineered factor that displays marked anti-apoptotic and pro-differentiative clues on muscle precursors. Its ability to promote and enhance muscle regeneration makes it a potential candidate molecule for regenerative medicine, particularly for muscular dystrophy syndromes and other muscle degenerative disorders. Given the small size of its cDNA (approximately 1 kb), Magic-F1 may be used alone in a gene therapy setting or inserted as a second adjuvant gene in a vector already encoding a therapeutic gene, for example encoding a deacetylase inhibitor [52]. The lack of mitogenic activity allows a safe use of Magic-F1 as a therapeutic cytokine, promoting muscle regeneration without the potential risk of stimulating uncontrolled proliferation.

## Materials and Methods

### Magic-F1 Factor engineering and purification

Magic-F1 is an engineered factor containing two HGF NK2 domains joint by a linker. The exact amino acidic sequence of Magic-F1 corresponds to: residues 1–285 of human HGF (Gene Bank # M73239); a linker with the sequence (GGGGS)_3_; residues 30–285 of human HGF; a poly-histidine tag with the sequence DDDKHHHHHH. Factors were produced in a CHO cell line (ATTC, Rockville, Maryland). Purification was performed by dual-step affinity chromatography using a heparin-Sepharose column and a Ni^2+^-chelate column (Amersham Pharmacia, Uppsala, Sweden). Activated human recombinant HGF was purchased from R&D Systems (Minneapolis, Minnesota) while Metron Factor-1 (Metr. in Figure S1) recombinant protein [53] was produced at Dompé Pharmaceutical Company S.p.A. (L'Aquila, Italy).

### ELISA binding assay

HGF and FcMET (a chimera consisting of the extracellular domain of MET fused to the Fc region of a human IgG_1_) were purchased from R&D Systems. Binding of Magic-F1 and HGF to Fc-Met was measured by ELISA using the receptor in solid phase and the ligands in liquid phase. A fixed concentration (100 ng/well) of Fc-Met was adsorbed to 96-well ELISA plates and incubated with increasing concentrations of ligands. Binding was revealed using biotinylated anti-HGF antibodies (R&D). Binding data were analyzed and fit using Prism software (Graph Pad Software, San Diego, California).

### Immunoreagents

The antibodies used in this study were obtained as follows: anti-human HGF for both Western blotting and immunoprecipitation, Santa Cruz Biotechnology (Santa Cruz, California); anti-human Met for Western blotting, Santa Cruz; anti-human Met for immunoprecipitation, as described [5]; anti-mouse Met, Santa Cruz; anti-AKT and anti-phospho-AKT, New England Biolabs (Beverly, Massachusetts); anti-MAPK (p42–44/ERK) and anti-phospho-MAPK, Promega (Madison, Winsconsin); anti-laminin polyclonal rabbit antibodies, Sigma (St. Louis, Missouri); anti-desmin rabbit polyclonal antibody, Sigma; MF20 and embryonic myosin monoclonal antibody, Developmental Studies Hybridoma Bank (Iowa City, Iowa).

### Receptor activation and signal transduction

For receptor activation analysis, quiescent cells plated on collagen-coated 100 mm plates (Becton Dickinson, Franklin Lakes, New Jersey) were stimulated with 5 nM HGF or Magic-F1 for 30 min at 37°C and then lysed as described [54]. Lysates were immunoprecipitated with anti-Met and analyzed by Western blotting using anti-phosphotyrosine antibodies. For signal-transduction analysis, cells were stimulated as above for different times and then lysed. For MAPK and AKT activation, lysates were directly analyzed by Western blotting using antibodies specific for the activated forms of the signaling molecules. Quantification of enhanced chemiluminescence signal was performed using a STORM apparatus and Image Quant software (Molecular Dynamics, Amersham Biosciences, Sunnyvale, California).

### Cell cultures and bioassays

Mouse myogenic cell line C2C12 was maintained in DMEM supplemented with 2 mM glutamine, 100 IU/ml penicillin, 100 µg/ml streptomycin and 10% FBS. C2C12 cells were induced to differentiate into myotubes by replacing 10% FBS with 2% horse serum (HS). Differentiation was completed in 7–8 days. All cultures were performed at 37°C in a humidified incubator with 5% CO_2_ and 95% air. Satellite cells were prepared as previously described [46]. Briefly, muscle fragments were digested with 2% collagenase II (Invitrogen, Carlsbad, California) for 60 min at 37°C. Digested cells were discarded and fragments were incubated again with 0.05% trypsin (Invitrogen) for 15 min at 37°C with gentle agitation. After the incubation, isolated cells were collected and fragments were incubated again until the whole tissue was digested (usually three times). Isolated cells were pooled, centrifuged and resuspended in DMEM supplements with 20% pre-screened FCS, 1% gentamycin, and plated onto collagen coated dishes at a density of 10^4^ cells×cm^2^. Contamination by non-myogenic cell was reduced by pre-plating the cell suspension onto plastic dishes where fibroblasts tend to adhere more rapidly. Differentiation was induced shifting the medium to DMEM supplemented with 2% horse serum. Cell morphology was examined daily with a phase-contrast microscope connected to an image analyzer. Cells were trypsinized daily and counted on a hemocytometer. Cell viability was determined by trypan blue dye exclusion assay. Cell cytotoxicity was performed using an XTT-based *in vitro* toxicology assay kit (Sigma) according to manufacturer's protocol. Incubation medium was collected after 3 hours and read spectrophotometrically at a wavelength of 450 nm. Background signals, obtained from plates without cells, were subtracted from sample readings. Apoptosis was quantified using an ApopTag Fluorescein In situ Apoptosis detection kit (Chemicon, Temecula, California) according to the manufacturer's protocol. Cell differentiation was carried out for 8 days. Cells were grown on 6 cm Petri dishes until sub-confluent, washed with PBS, fixed with 4% paraformaldehyde at room temperature for 10 minutes and then permeabilized with 0.1% Triton X-100 in PBS for 5 minutes. After incubation with PBS containing 10% normal serum, samples were incubated overnight at 4°C with anti-GFP at 1∶200 dilution, anti myosin heavy chain (MF20) antibody at 1∶2 dilution. After incubation, cells were washed three times in PBS and incubated with the appropriate FITC- or TRITC-conjugated secondary antibodies for 1 hour at room temperature. After washing in PBS, cells were analyzed under a fluorescent microscope and photographed. As a control for the immunofluorescence method, we omitted the primary antibody and no staining was detected under these conditions. Cell nuclei were counterstained with DAPI.

### Plasmids and DNA preparation

Magic-F1 was cloned into pIRESneo (Clontech, Italy) for C2C12 transfection experiments whereas it was cloned into pcDNA3 (Invitrogen) containing the cytomegalovirus (CMV) promoter for electrotransfer experiments (pCMV-Magic-F1); a pCMV-bgal plasmid coding for beta-galactosidase and a pCMV-hHGF plasmid coding for human hepatocyte growth factor were also used. Plasmids were prepared by using standard procedures. All plasmid preparations was obtained using a GenElute™ HP Endotoxin-Free Plasmid Maxiprep Kit (Sigma) and contained a high percentage of supercoiled DNA (70–80%). No RNA was detectable by gel electrophoresis.

### DNA electro-transfer and animal handling

Mouse experiments were performed in the San Raffaele Hospital SPF Animal Care Facilities according to international ethical guidelines (EEC Council Directive 86/609; NIH Guide for the Care and Use of Laboratory Animals, 1985). Authorization for animal experimentation was obtained from the Italian Ministry of Health. Gene transfer into skeletal muscle mediated by electric pulse was performed as previously reported [33]. Briefly, 20 µg of DNA in 10 µl of PBS was injected into the *tibialis anterior* or in the *quadriceps* muscle of anesthetized, 10 day-old C57Bl/6 mice (Iffa Credo, St. Germain sur l'Arbresle, France) with a Hamilton syringe. There were 10 muscles included in each experimental group. Five minutes after DNA injection trans-cutaneous electric pulses were applied by two stainless steel plate electrodes placed 3.8–4.3 mm apart, at each side of the leg. Electrical contact with the leg skin was ensured by shaving each leg and applying a conductive gel. Square-wave electric pulses (eight pulses; 200 V/cm; 20 ms per pulse; 1 Hz) were generated by a digital Stimulator (Panlab 3100, Biological Instruments, Varese, Italy).

### Muscular regeneration analysis

Acute skeletal muscle damage was induced in male and female MLC1F/Magic-F1transgenic mice and control mice (7 animals/group) by i.m. injection of 10 nM cardiotoxin (Gentaur, Brussels, Belgium) in physiologic solution (0.9% w/v NaCl). Control mice were injected with physiologic solution alone. At 3, 7, and 14 days after drug injection, mice were sacrified and subjected to histological evaluation and morphometric analysis of *tibialis anterior*. After excision, muscles were sectioned (4–6 µm) and processed for immunofluorescence analysis using the primary antibodies listed above. All sections were washed three times in PBS and incubated with 10% donkey serum for 30 min at RT before the addition of the appropriate Alexa 488-, Alexa 594- or Alexa 647-conjugated donkey secondary antibodies. Alternatively, some sections were stained with hematoxylin and eosin and examined by an independent histopathologist not informed of sample identity to determine muscle fiber sizes using Scion Image software (Scion, Frederick, Maryland).

### Biochemical and molecular analysis

Western blot analysis of cells or tissues was performed as described [41], 55. Total RNA from control or treated cells was extracted using Trizol reagent (Invitrogen) and analysed by PCR after reverse transcription with random hexamers. RT-PCR analysis has been performed using the following primers:

Bax
Fw 5′-TGTTTGCTGATGGCAACTTC-3′
Rv 5′-GATCAGCTCGGGCACTTTAG-3′
Bcl-2
Fw 5′-GGGATGCCTTTGTGGAACTA-3′
Rv 5′-CTCACTTGTGGCCCAGGTAT-3′
P53
Fw 5′-GGATGCCCGTGCTGCCGAGGAG-3′
Rv 5′-AGTGAAGGGAC TAGCATTGTC-3′
Magic-F1
Fw 5′-TTCAGAAGTTGAATGCATGACCTG-3′
Rv 5′-TCTTCTTTTCCTTTGTCCCTCTAG-3′
GAPDH
Fw 5′-TTCACCACCATGGAGAAGGC-3′
Rv 5′-GGCATGGACTGTGGTCATGA-3′
MyoD
Fw 5′-TGCACTTCCACCAACCCCAACCAGC-3′
Rv 5′-CCTGGACTCGCGCACCGCCTCACT-3′
Met
Fw 5′-AGAAATTCATCAGGCTGTGAAGCGCG-3′
Rv 5′-TTCCTCCGATCGCACACATTTGTCG-3′
PAX3
Fw 5′-AGGAGGCGGATCTAGAAAGGAAG-3′
Rv 5′-TGTGGAATAGACGTGGGCTGGTA-3′
Myf5
Fw 5′-GAGCTGCTGAGGGAACAGGTGGAGA-3′
Rv 5′-GTTCTTTCGGGACCAGACAGGGCTG-3′
IGF1
Fw 5′-CTGTGCCCCACTGAAGCCTA-3′
Rv 5′-GGACTTCTGAGTCTTGGGCATG-3′
Myostatin
Fw 5′-AGTGACGGCTCTTTGGAAGATG-3′
Rv 5′-AGTCAGACTCGGTAGGCATGGT-3′
Follistatin
Fw 5′-CTGTACAAGACCGAACTGAGC-3′
Rv 5′-TCCACAGTCCACGTTCTCACA-3′

### Generation of Magic-F1 and α-SG knock-out/Magic-F1 transgenic mice

We constructed the transgene by inserting the Magic-F1 construct into the pMex plasmid containing the 1,500-bp fragment of the MLC promoter, an 840-bp fragment of SV40 poly(A), and a 900-bp fragment from the 3′ end of the MLC1f/3f gene, which acts as an enhancer [34]; provided by Dr. Antonio Musarò, University of Rome, Italy. We microinjected the transgene into the male pronucleus of fertilized eggs from FVB mice (Jackson Laboratories, Bar Harbor, Maine) that were implanted into pseudopregnant foster mothers. We identified positive transgenic mice by PCR. For PCR detection, sense and antisense primers specific respectively for the MLC1F promoter and the linker region of Magic-F1 were used. Transgenic founders were mated with wild-type FVB mice to generate F1 offspring. After obtaining MLC1F/Magic-F1 mice we mated them with α-SG knock-outs [36] to generate α-SG knock-out/Magic-F1 transgenic mice. The animals were housed in a temperature controlled (22°C) room with a 12∶12 hours light-dark cycle. All studies have been performed using Tg:MLC1F/Magic-F1 hemizygous mice, following the protocols approved by the Animal Care and Use Committee of the San Raffaele Institute (IACUC 264) and communicated to the Ministry of the Health and local authorities according to Italian law.

### Adenovirus preparation and administration

pAd/CMV-Magic-F1/V5-DEST was engineered using the ViraPower Adenoviral Expression System from Invitrogen. The Adenoviral vector was linearized with *Pac*I restriction enzyme and transfected into 293A cells. Cells were grown in Iscove Medium supplemented with 10% heat-inactivated FBS, 2 mM l-glutamine, 50 units/ml penicillin, 50 µg/ml streptomycin (Sigma). After complete detachment of cells, the supernatant was used to superinfect 293A cells. The purification of Adenoviral particles was performed with Vivapure AdenoPACK 100TM (Sartorius, Goettingen, Germany) starting from 200 ml of cell culture. Juvenile α-SG knock-out mice (8 weeks old) were anesthetized with an intraperitoneal injection of avertin (0.2 ml/10 g bodyweight of a 1.2% solution), hair was shaved from the skin and pAd-Magic-F1 suspension (2.5×10^9^ pfu diluited in 30 µl of PBS containing 100 ng of VEGF) were injected i.m. with a 30-gauge needle in the center of *gastrocnemius*, *quadriceps* and *tibialis anterior*. To prevent an immune-mediated clearance of adeno-infected fibers, all mice were immunosuppressed with FK506 (5 mg/day/Kg, subcutaneously). The immunosuppressive treatment was started on the day before the Adenoviral injection and continued until mice were sacrificed.

### Treadmill analyses

Treadmill analyses were carried out using a six-lane motorized treadmill (Exer 3/6 Treadmill; Columbus Instruments, Columbus, Ohio) supplied with shocker plates. The first trial was performed at low intensity and for short duration to accustom the mice to the exercise (5 m/min for 5 minutes, after which the speed was increased 1 m/min every 2 minutes until it reached 9 m/min). After the first trial, the treadmill was run at an inclination of 0° at 5 m/min for 5 minutes, after which the speed was increased 1 m/min every 1 minute. The test was stopped when the mouse remained on the shocker plate for more than 20 s without attempting to re-engage the treadmill, and the time to exhaustion was determined.

## Supporting Information

Figure S1 Production of Magic-F1 in eucaryotic systems. (A) After transient transfection with pIRES-neo-Magic-F1 plasmid, cells were washed and incubated with fresh serum-free medium; aliquots of medium after 3, 6 and 18 hours (lane 1, 2 and 3, respectively) were concentrated 100 times and subjected to Western blot analysis using anti-HGF antibodies. Cell lysate (lane 5) and mock conditioned medium (lane 4) were also analyzed. (B) Growth curves of CHO cells expressing Magic-F1 (#PM21) or transfected with an empty vector (CHO). (C) Western blot analysis of conditioned media from different CHO clones expressing Magic-F1 at different levels. (D) Quantification of Magic-F1 protein in the conditioned medium of clone # 21; a related recombinant protein, Metron Factor-1, was used as standard (Metr). (E) Immunoprecipitation of Magic-F1 protein from the conditioned medium of clone # 5, 7, 21 and 25. In some case (# 7f), following storage at −20°C, Magic-F1 could not be immunoprecipitated any more. In each lane we loaded 10 µl of medium concentrated 100 times.
(0.73 MB TIF)

Figure S2 Quantitative RT-PCR analysis of Magic-F1-transfected C2C12 cells. The expression of 36 genes involved in myogenic differentiation was evaluated one day after transfection of C2C12 using quantitative real time PCR analysis. The results indicate that 30 out of the 36 analyzed genes were upregulated in C2C12 expressing Magic-F1 compared to controls, confirming an enhanced rate of differentiation induced by Magic-F1 expression.
(0.54 MB TIF)

Figure S3 Magic-F1 enhances muscle regeneration. (A) Morphometric analysis on a tibialis anterior section shows a marked increase of the fiber area in Magic-F1 transgenic mice (red bar) relative to wild-type mice (blue bar). Note that the number of fibers with a larger cross sectional area is higher in Magic-F1 transgenic mice when compared to wild-type mice. This effect is evident in both regenerated and regenerating fibers (centrally nucleated) as showed in (B), where statistical analysis is reported.
(1.11 MB TIF)
