# Correction: Magic-Factor 1, a Partial Agonist of Met, Induces Muscle Hypertrophy by Protecting Myogenic Progenitors from Apoptosis

**DOI:** 10.1371/journal.pone.0220357

**Published:** 2019-07-24

**Authors:** Marco Cassano, Stefano Biressi, Amanda Finan, Laura Benedetti, Claudia Omes, Renata Boratto, Frank Martin, Marcello Allegretti, Vania Broccoli, Gabriella Cusella De Angelis, Paolo M. Comoglio, Cristina Basilico, Yvan Torrente, Paolo Michieli, Giulio Cossu, Maurilio Sampaolesi

After publication of this article [1], the following concerns were raised about the figures:

- Figure 2E follistatin and GAPDH panels contain background irregularities such that lane 1 appears to have a different background to lanes 2–6;
- Figure 3B Magic-F1 and HGF panels contain background irregularities such that lanes 1–3 appear to have a different background to lanes 4–6;
- Fig 5A WT 3d and WT 7d panels appear similar.

Regarding Fig 5A, the authors clarify that the bottom images at 3d and 7d for WT have been erroneously swapped. In the published paper the image below 3d should be below 7d, and vice versa. This is a double IF analysis to test embryonic myosin (red) and laminin (green). Thus all WT upper panels are same images of below using a different light channel.

The authors have provided the original images used in Figures 2E and 3B. The original images do not contain background irregularities and the source of the image artifact is unclear.

With this correction, the authors provide an updated Fig 5, along with the original images underlying Figs 5A, 2E, and 3B (in Supporting Information S1 File).

**Fig 5:**
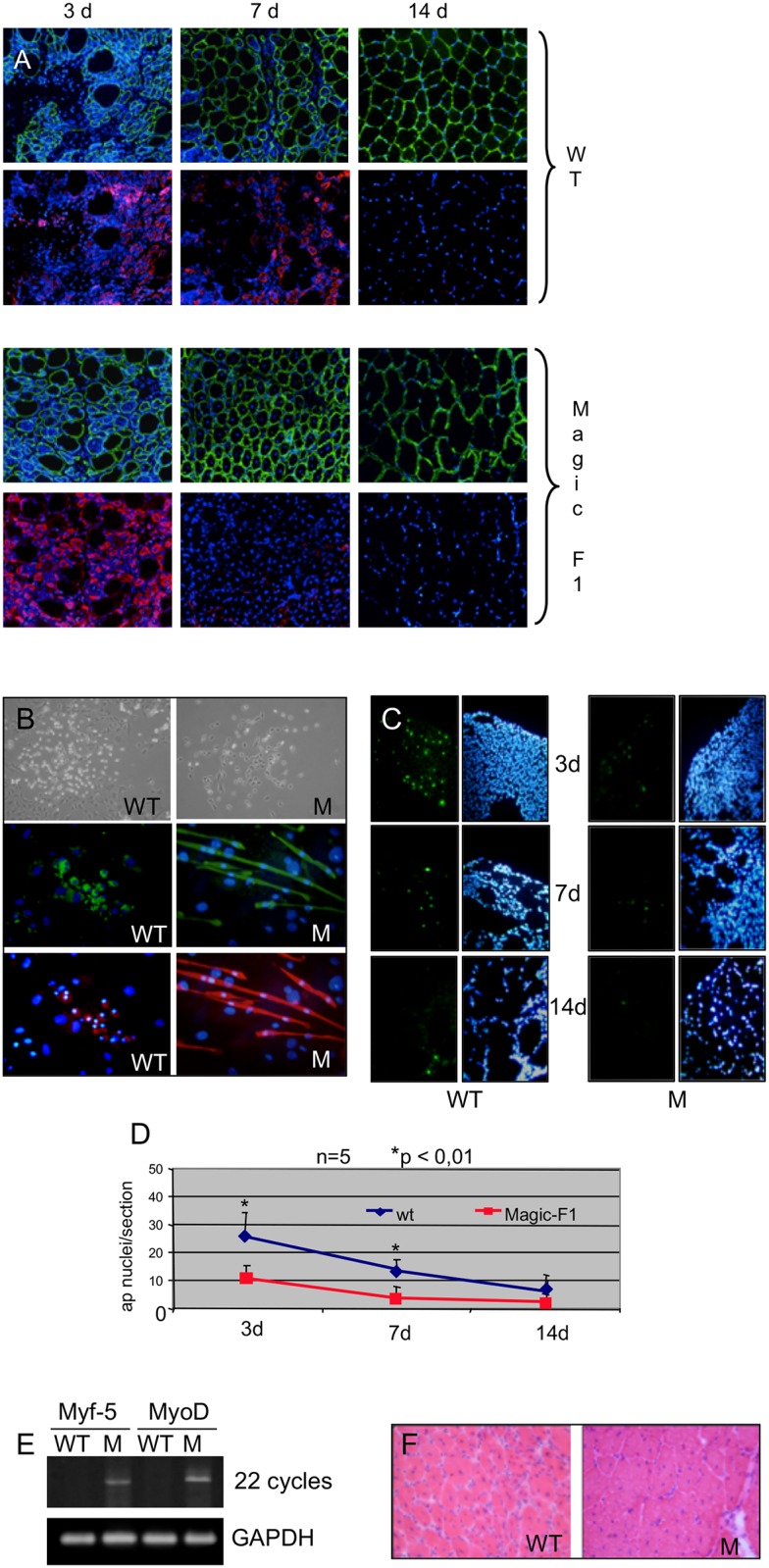
Magic-F1 promotes muscular regeneration. (A) Immunofluorescence analysis of muscle fibers using antibodies against embryonic myosin heavy chain (red) or laminin (green) in the *tibialis anterior* of transgenic and wild-type mice. Nuclei were stained with DAPI. (B) Immunofluorescence analysis for desmin (middle panels, in green) and myosin heavy chain (lower panels, in red) of satellite cells isolated from *tibialis anterior* of Magic-F1 transgenic mice (M) and wild-type (WT) mice subjected to cardiotoxin treatment. Nuclei are stained with DAPI (in blue). The upper panels show a phase contrast image of satellite cell clones, 3 days after low density seeding. (C) TUNEL analysis of *tibialis anterior* after 3, 7 and 14 days after cardiotoxin treatment. (D) Quantification of apoptotic nuclei (ap nuclei) relative to the experiment described in C. Red line, transgenic mice; blue line, wild-type mice. (E) RT-PCR analysis of myogenic transcription factor expression (MyoD and Myf5) conducted on *tibialis anterior* from transgenic (M) or wild-type (WT) mice. (F) Representative images of *tibialis anterior* muscles stained with H&E extracted from Magic-F1 transgenic mice and wild-type mice 10 days after cardiotoxin treatment. Note the larger size of fibers in the Magic-F1 group (M) compared to the control group (WT).

## Supporting information

S1 File Original raw images for Figs 5A, 2E, and 3B. (ZIP)
