# Supplementary figures and images for: Magic-Factor 1, a Partial Agonist of Met, Induces Muscle Hypertrophy by Protecting Myogenic Progenitors from Apoptosis
Source: PLoS One. 2008 Sep 16;3(9):e3223. doi: 10.1371/journal.pone.0003223 (PMC2528937; doi:10.1371/journal.pone.0003223)

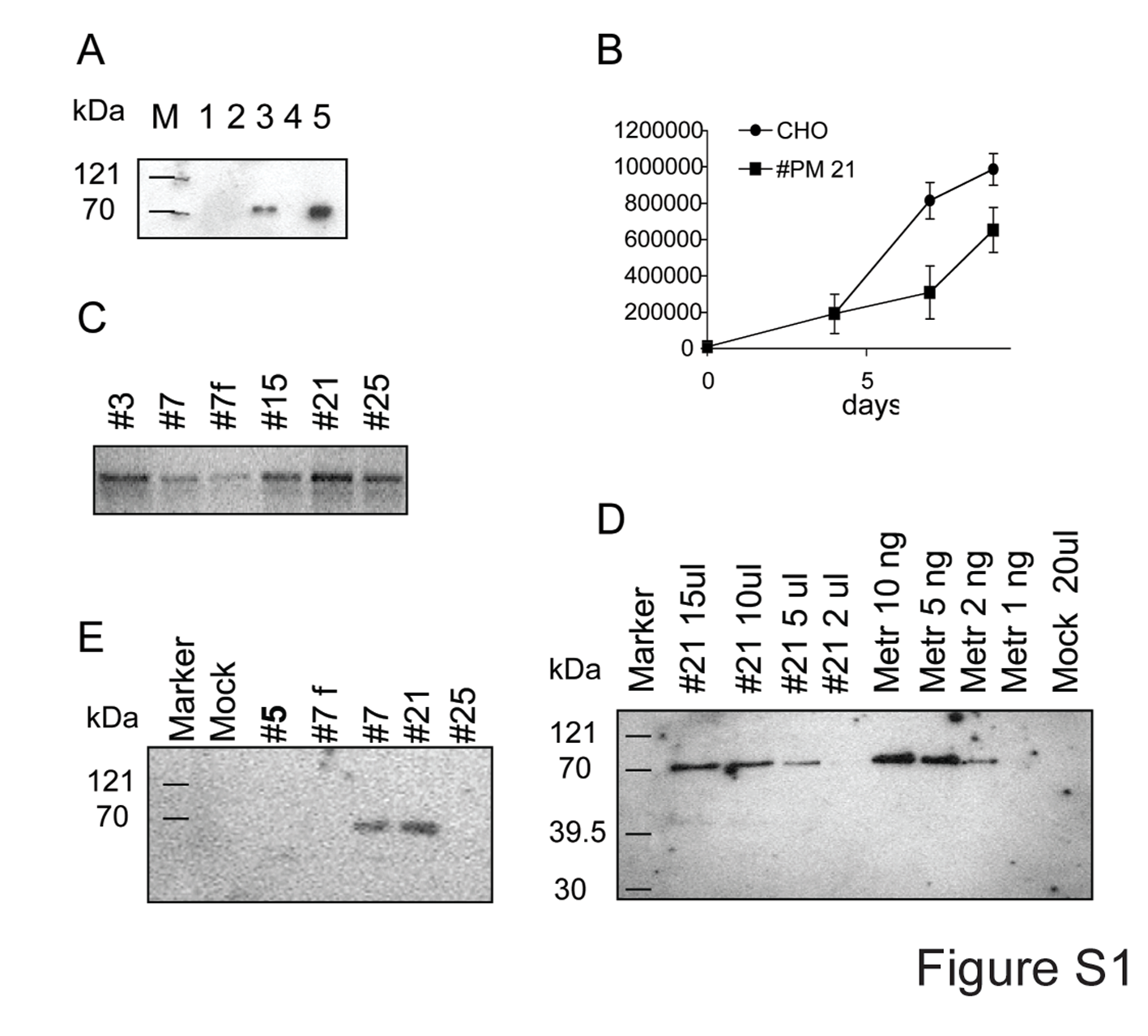

Supplement: Figure S1 — Production of Magic-F1 in eucaryotic systems. (A) After transient transfection with pIRES-neo-Magic-F1 plasmid, cells were washed and incubated with fresh serum-free medium; aliquots of medium after 3, 6 and 18 hours (lane 1, 2 and 3, respectively) were concentrated 100 times and subjected to Western blot analysis using anti-HGF antibodies. Cell lysate (lane 5) and mock conditioned medium (lane 4) were also analyzed. (B) Growth curves of CHO cells expressing Magic-F1 (#PM21) or transfected with an empty vector (CHO). (C) Western blot analysis of conditioned media from different CHO clones expressing Magic-F1 at different levels. (D) Quantification of Magic-F1 protein in the conditioned medium of clone # 21; a related recombinant protein, Metron Factor-1, was used as standard (Metr). (E) Immunoprecipitation of Magic-F1 protein from the conditioned medium of clone # 5, 7, 21 and 25. In some case (# 7f), following storage at −20°C, Magic-F1 could not be immunoprecipitated any more. In each lane we loaded 10 µl of medium concentrated 100 times. (0.73 MB TIF) [file pone.0003223.s001.tif]

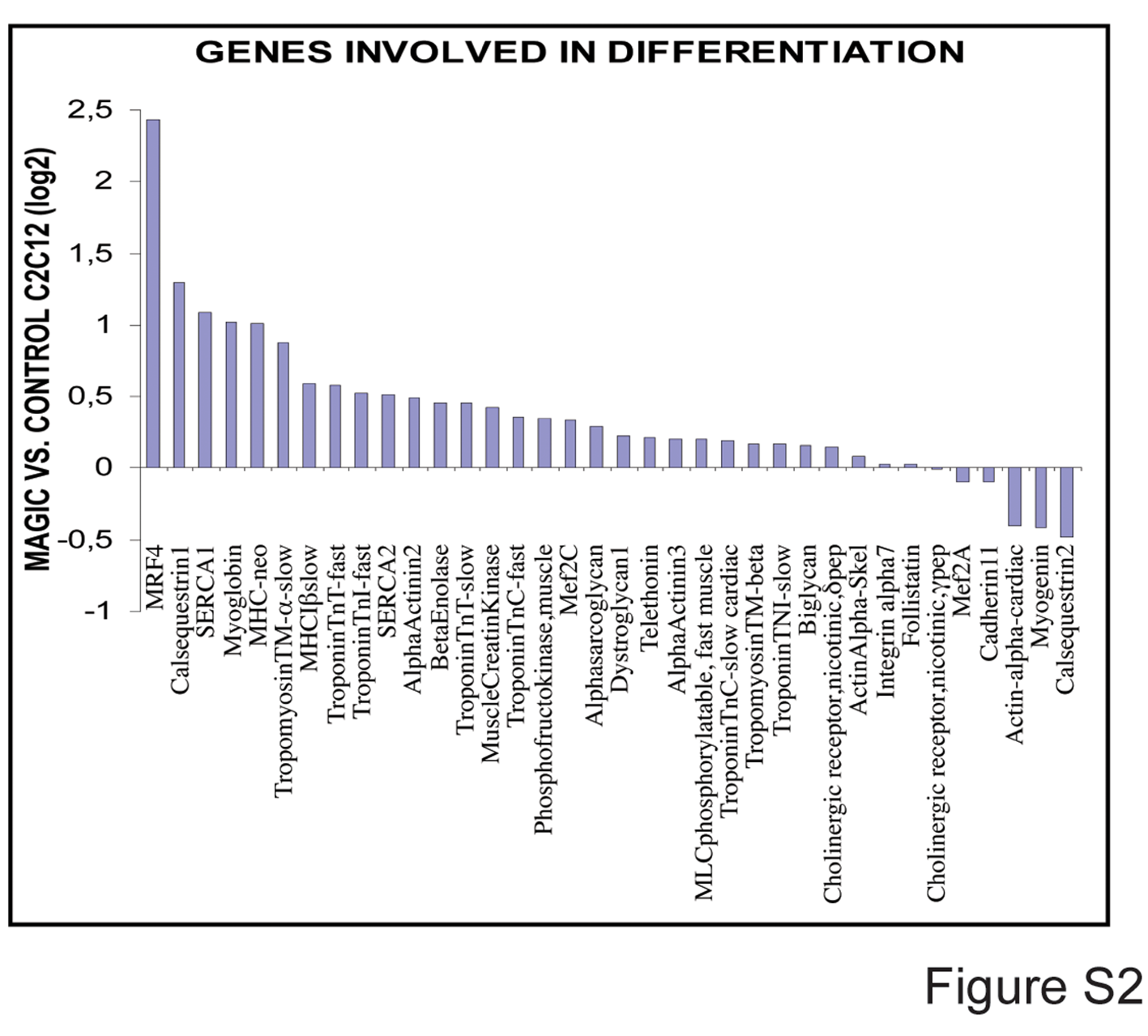

Supplement: Figure S2 — Quantitative RT-PCR analysis of Magic-F1-transfected C2C12 cells. The expression of 36 genes involved in myogenic differentiation was evaluated one day after transfection of C2C12 using quantitative real time PCR analysis. The results indicate that 30 out of the 36 analyzed genes were upregulated in C2C12 expressing Magic-F1 compared to controls, confirming an enhanced rate of differentiation induced by Magic-F1 expression. (0.54 MB TIF) [file pone.0003223.s002.tif]

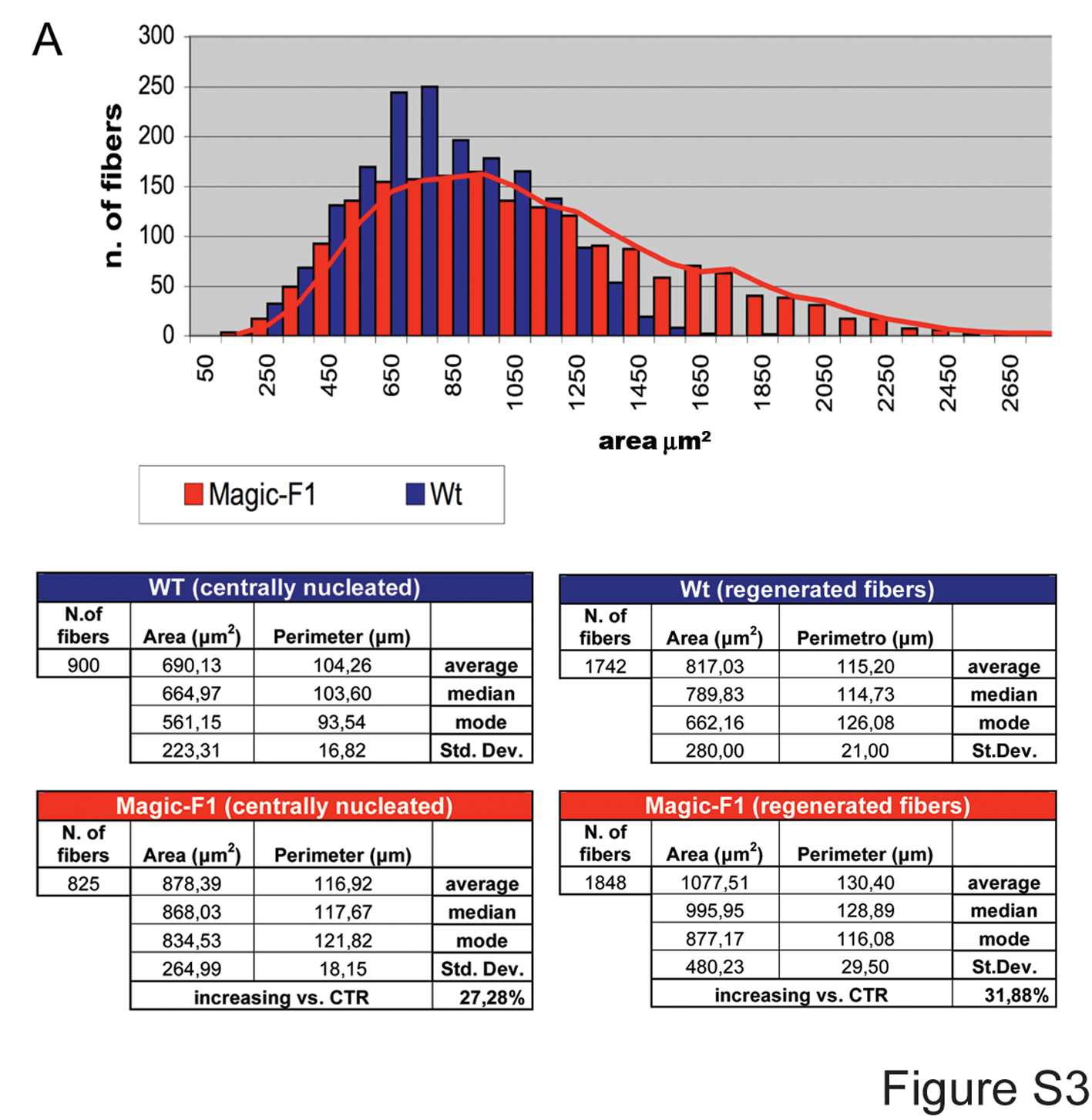

Supplement: Figure S3 — Magic-F1 enhances muscle regeneration. (A) Morphometric analysis on a tibialis anterior section shows a marked increase of the fiber area in Magic-F1 transgenic mice (red bar) relative to wild-type mice (blue bar). Note that the number of fibers with a larger cross sectional area is higher in Magic-F1 transgenic mice when compared to wild-type mice. This effect is evident in both regenerated and regenerating fibers (centrally nucleated) as showed in (B), where statistical analysis is reported. (1.11 MB TIF) [file pone.0003223.s003.tif]
