# Supplementary figures and images for: Correction: Magic-Factor 1, a Partial Agonist of Met, Induces Muscle Hypertrophy by Protecting Myogenic Progenitors from Apoptosis
Source: PLoS One. 2019 Jul 24;14(7):e0220357. doi: 10.1371/journal.pone.0220357 (PMC6655771; doi:10.1371/journal.pone.0220357)

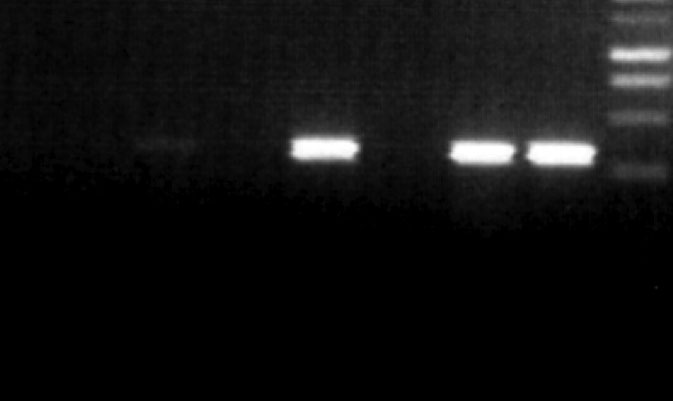

Supplement: S1 File — (ZIP) [file pone.0220357.s001.zip › S1 File/Fig2E Myostatin panel.jpg]

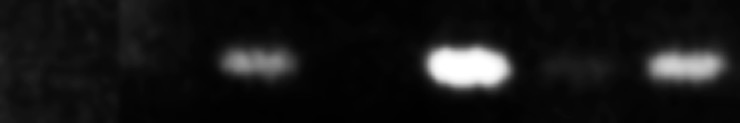

Supplement: S1 File — (ZIP) [file pone.0220357.s001.zip › S1 File/Fig2E Follistatin.jpg]

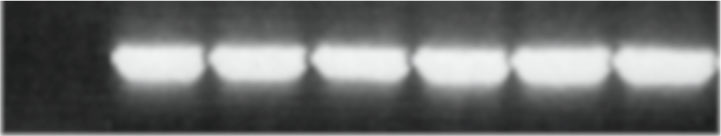

Supplement: S1 File — (ZIP) [file pone.0220357.s001.zip › S1 File/Fig2E GAPDH panel.jpg]

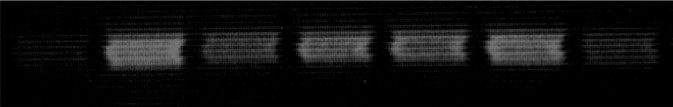

Supplement: S1 File — (ZIP) [file pone.0220357.s001.zip › S1 File/Fig2E IGF1 panel.jpg]

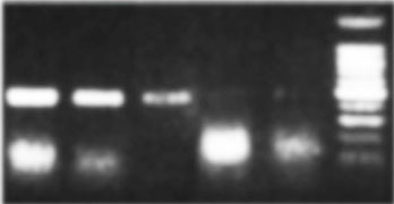

Supplement: S1 File — (ZIP) [file pone.0220357.s001.zip › S1 File/Fig3B HGF panel.jpg]

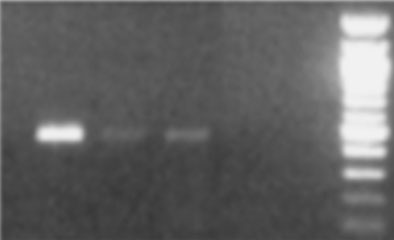

Supplement: S1 File — (ZIP) [file pone.0220357.s001.zip › S1 File/Fig3B MagicF1 panel.jpg]

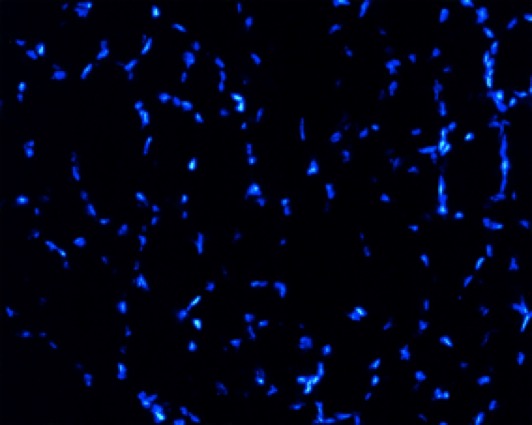

Supplement: S1 File — (ZIP) [file pone.0220357.s001.zip › S1 File/MAGIC-F1 14d eMyHC Fig5A.jpg]

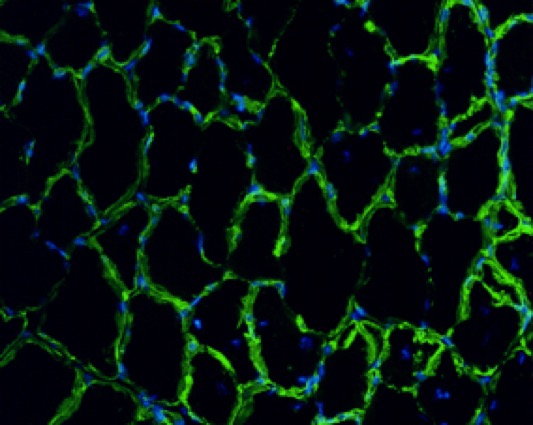

Supplement: S1 File — (ZIP) [file pone.0220357.s001.zip › S1 File/MAGIC-F1 14d laminin Fig5A.jpg]

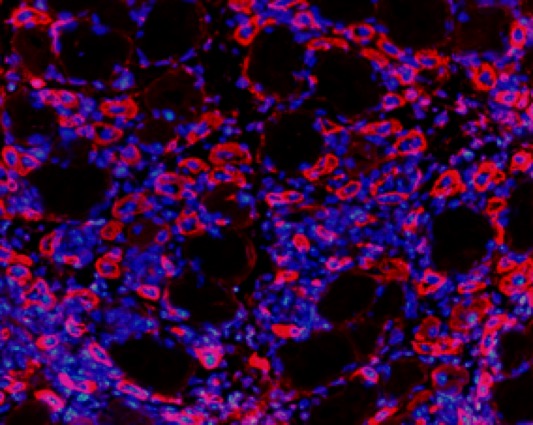

Supplement: S1 File — (ZIP) [file pone.0220357.s001.zip › S1 File/MAGIC-F1 3d eMyHC Fig5A.jpg]

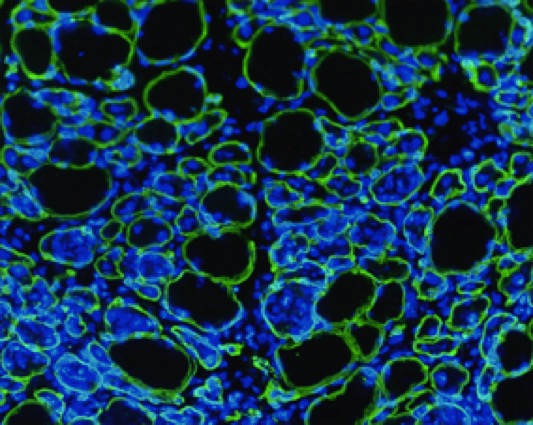

Supplement: S1 File — (ZIP) [file pone.0220357.s001.zip › S1 File/MAGIC-F1 3d laminin Fig5A.jpg]

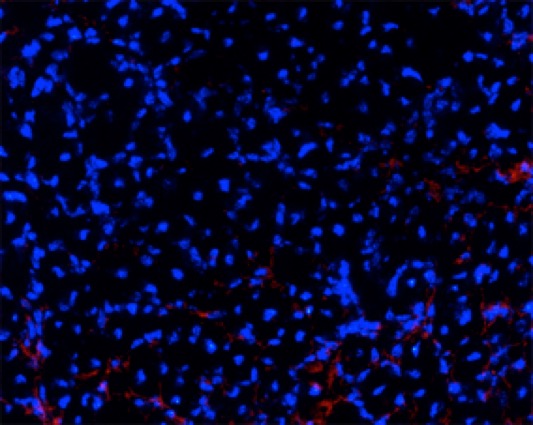

Supplement: S1 File — (ZIP) [file pone.0220357.s001.zip › S1 File/MAGIC-F1 7d eMyHC Fig5A.jpg]

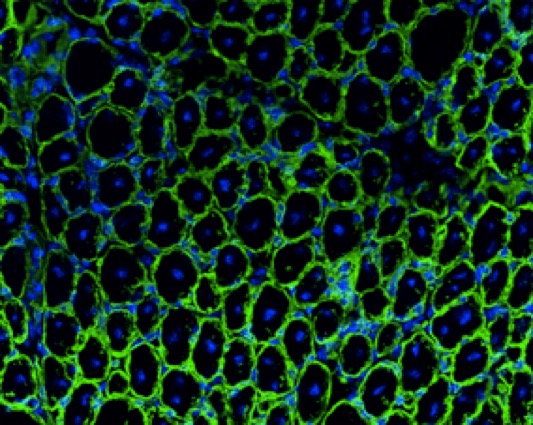

Supplement: S1 File — (ZIP) [file pone.0220357.s001.zip › S1 File/MAGIC-F1 7d laminin Fig5A.jpg]

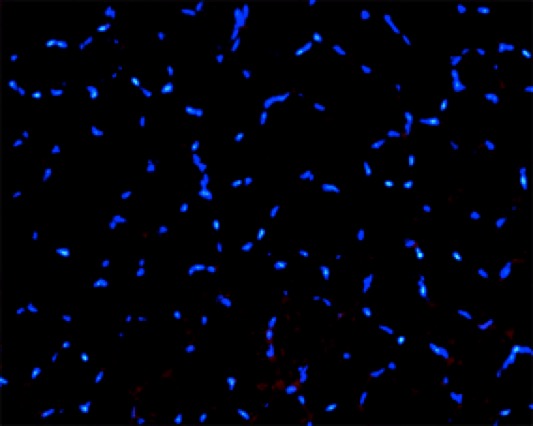

Supplement: S1 File — (ZIP) [file pone.0220357.s001.zip › S1 File/WT 14d eMyHC Fig5A.jpg]

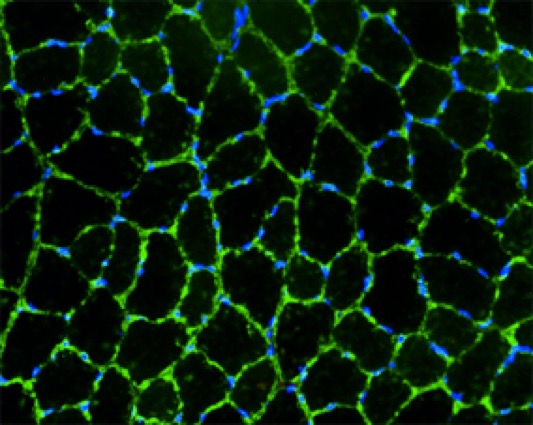

Supplement: S1 File — (ZIP) [file pone.0220357.s001.zip › S1 File/WT 14d laminin Fig5A.jpg]

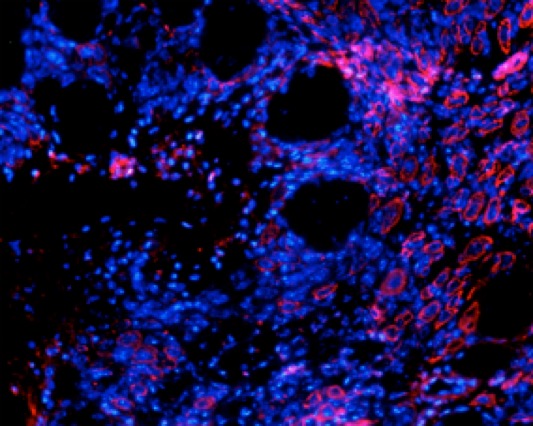

Supplement: S1 File — (ZIP) [file pone.0220357.s001.zip › S1 File/WT 3d eMyHC Fig5A.jpg]

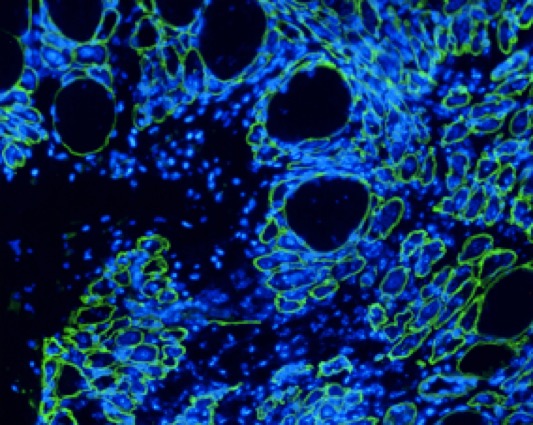

Supplement: S1 File — (ZIP) [file pone.0220357.s001.zip › S1 File/WT 3d laminin Fig5A.jpg]

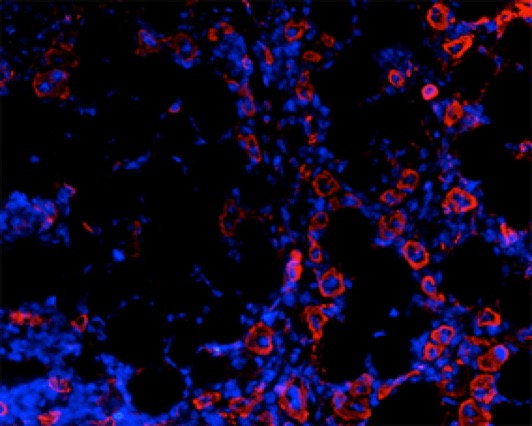

Supplement: S1 File — (ZIP) [file pone.0220357.s001.zip › S1 File/WT 7d eMyHC Fig5a.jpg]

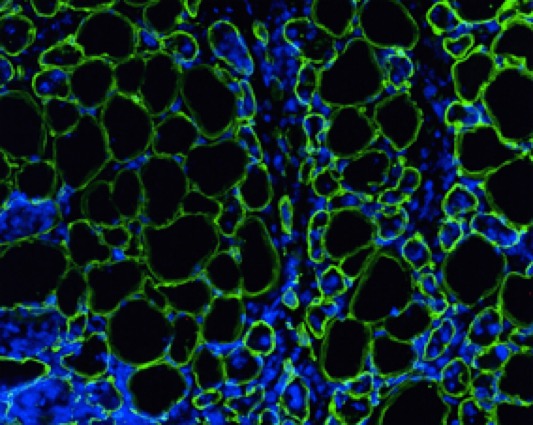

Supplement: S1 File — (ZIP) [file pone.0220357.s001.zip › S1 File/WT 7d laminin Fig5A.jpg]
